# Tortuous extracranial arteries contribute to white Matter hyperintensities in aging brains

**DOI:** 10.3389/fnagi.2025.1641214

**Published:** 2025-10-09

**Authors:** Zhe Sun, Chenyang Li, Arjun V. Masurkar, Marco Muccio, Thomas Wisniewski, Yulin Ge

**Affiliations:** ^1^Department of Radiology, NYU Grossman School of Medicine, New York, NY, United States; ^2^Vilcek Institute of Graduate Biomedical Sciences, NYU Grossman School of Medicine, New York, NY, United States; ^3^Center for Cognitive Neurology, Department of Neurology, NYU Grossman School of Medicine, New York, NY, United States; ^4^Department of Neuroscience and physiology, NYU Grossman School of Medicine, New York, NY, United States; ^5^Department of Pathology, NYU Grossman School of Medicine, New York, NY, United States; ^6^Department of Psychiatry, NYU Grossman School of Medicine, New York, NY, United States

**Keywords:** white matter hyperintensity, internal carotid artery, vertebral artery, tortuosity, blood flow, magnetic resonance angiography

## Abstract

**Introduction:**

White matter hyperintensity (WMH) is a hallmark imaging biomarker of cerebral small vessel disease and are strongly associated with vascular cognitive impairment in the elderly. Morphological changes in large extracranial brain-feeding arteries, such as the internal carotid (ICA) and vertebral arteries (VA), may alter intracranial hemodynamics and contribute to WMH development. This study examined the relationship between arterial tortuosity and WMHs using magnetic resonance angiography (MRA).

**Methods:**

Seventy-eight participants underwent time-of-flight (TOF) MRA and phase-contrast (PC) MRI to assess arterial morphology and blood flow. After excluding three for poor image quality, 75 subjects were analyzed. Arterial tortuosity was quantified using the inflection count metric (ICM) and ICA angle. Global cerebral blood flow (CBF) was estimated with PC-MRI and compared against pseudo-continuous arterial spin labeling (pCASL) to determine whether it could be a reliable surrogate measurement to reflect intracranial blood supply.

**Results:**

Participants with severe WMHs (Fazekas ≥2) demonstrated greater tortuosity (higher ICM and larger ICA angles) and lower blood flow than those with mild WMHs. Females showed more tortuous arteries, greater WMH burden, and higher susceptibility to hypoperfusion. Correlation analyses revealed a positive association between tortuosity and WMH volume.

**Discussion:**

These findings highlight the role of extracranial arterial tortuosity in WMH burden and reveal sex-specific differences in vascular vulnerability. The results underscore the need for further investigation into how age-related vascular remodeling contributes to WMH development and cognitive decline.

## 1 Introduction

White matter hyperintensities (WMHs) are frequently observed on T2-weighted and fluid-attenuated inversion recovery (FLAIR) magnetic resonance imaging (MRI) in the elderly. They serve as imaging markers of cerebral small vessel disease (SVD) and have been linked to cognitive impairment and progression along the dementia spectrum (Bangen et al., 2018; Morrison et al., 2022; Courtney et al., 2025). Among various factors that may contribute to WMH development, such as vascular abnormalities, metabolic disorders, inflammation, and toxins (Wu et al., 2021), ischemic changes are widely recognized as the primary underlying cause. Proposed mechanisms, such as endothelial dysfunction and disruption of the blood-brain barrier, play a role in WMH pathogenesis but do not fully account for complexity of the underlying processes (Groh and Simons, 2025).

Large extracranial brain-feeding arteries, such as the internal carotid artery (ICA) and vertebral artery (VA), play a critical role in supplying blood and oxygen to the brain. Under normal physiological conditions, their elastic properties effectively modulate the pulsatile cardiac output and help ensure adequate downstream perfusion. With aging, however, vascular remodeling occurs, characterized by elastin degradation and collagen aggregation, ultimately leading to reduced arterial elasticity and increased tortuosity, manifesting as twisting and bending. These structural changes further impair the regulation of downstream pulse pressure, potentially disrupting distal microvascular networks and leading to tissue ischemia (Cocciolone et al., 2018; Robert et al., 2025). Histopathological studies have linked tortuous cerebral penetrating arteries to ischemic WMHs (Akashi et al., 2017; Hiroki et al., 2002). More recent, imaging studies employing ultrasound, computed tomography (CT), and MRI have identified geometric alterations in major arteries, including ICA and VA, among individuals with WMHs (Chen et al., 2020; Zhang et al., 2019). While the effects of intracranial and extracranial artery stenosis or occlusion on stroke are well documented, the role of extracranial arterial tortuosity in WMH pathogenesis remains underexplored, particularly in relation to cerebral blood flow.

Additionally, sex differences in aging and dementia have gained increasing attention in recent years. Previous studies have identified notable sex-related differences in both Alzheimer’s disease (AD) and vascular cognitive impairment, with women generally exhibiting higher incidence rates and faster disease progression than men (Chene et al., 2015; Pavlovic et al., 2022). The precise mechanisms, however, are not yet fully understood.

In this study, we aimed to: (1) assess whether metrics derived from time-of-flight (TOF) and phase-contrast (PC) MR angiography (MRA) can reliably characterize vessel properties, including geometry and flow; (2) investigate the relationships among vascular tortuosity, blood flow, and WMH severity in the elderly; (3) examine whether these vascular measurements differ by sex; and (4) compare the tortuosity effects on the cerebral blood flow (CBF) derived PC-MRI and pseudocontinuous arterial spin labeling (pcASL)-MRI. We hypothesized that increased tortuosity may alter intracranial blood supply, potentially contributing to WMH burden. Moreover, females may exhibit more tortuous vessels, which could influence downstream perfusion and lesion severity.

## 2 Materials and methods

### 2.1 Participants

**FIGURE 1:**
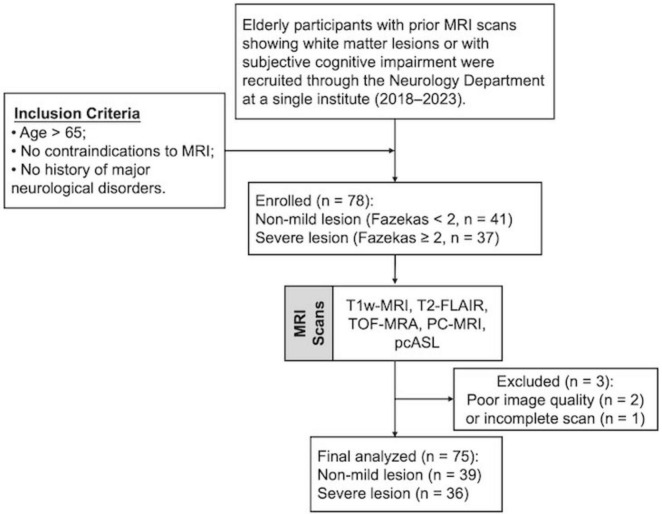
Flowchart of participant recruitment. FLAIR, fluid attenuation inversion recovery; TOF, time of flight; PC, phase contrast; ASL, arterial spin labeling.

Elderly participants with either prior MRI scans showing white matter lesions or subjective cognitive impairment were primarily recruited through referrals from the Department of Neurology between 2018 and 2023. The inclusion criteria were as follows: (1) age >65 years to minimize large age variance; (2) no contraindications to MRI (e.g., pacemakers, metallic implants, claustrophobia); and (3) no history of major neurological disorders, including stroke, traumatic brain injury, tumors, multiple sclerosis, or clinically diagnosed Alzheimer’s disease (AD). A total of 78 participants were enrolled in this prospective study (mean age: 73.1 ± 5.3 years; 42 females, 36 males). Three participants were excluded due to bad image quality or incomplete scans, leaving 75 participants for the final analysis (Figure 1). Body mass index (BMI) was recorded for all participants, and systolic and diastolic blood pressure (BP) was measured in 65 of the 75 participants prior to scanning. WMHs were visually rated on FLAIR MRI using Fazekas scores (Fazekas et al., 1987) by three independent raters, each with more than 5 years of experience. Participants were then categorized into two groups based on WMH severity: a mild group (Fazekas scores <2) and a severe group (Fazekas scores ≥2). Written informed consent was obtained from all participants, and the study protocol was approved by the NYU Grossman School of Medicine Institutional Review Board.

### 2.2 MR imaging acquisition

**FIGURE 2:**
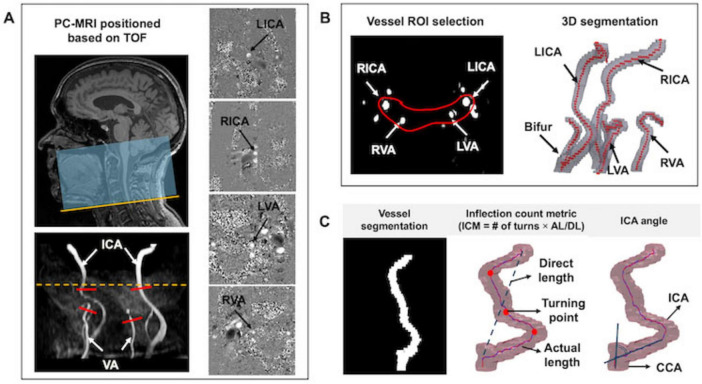
Image acquisition and postprocessing. **(A)** The TOF-MRA covered the region extending from the cervical vertebra C4 (yellow line) to the bottom of the pons. Four PC MRI scan planes (red bars) were positioned perpendicular to the major feeding arteries (i.e., LICA, RICA, LVA, and RVA), based on TOF maximum intensity projection (MIP) image. Meanwhile, the artery of interest was centered in the FOV. The yellow dash line represents the level of foramen magnum. Corresponding phase images are displayed in the right column. **(B)** ROI enclosing target arteries was drawn for vessel segmentation; **(C)** Tortuosity measurements, including ICM and ICA angle.

MRI scans were performed on a 3T scanner equipped with a 64-channel head coil. The imaging protocols and parameters were as follows: (1) T1-weighted magnetization-prepared rapid acquisition gradient echo (T1w-MPRAGE): repetition time (TR)/echo time (TE)/flip angle (FA) = 2100 ms/4.18 ms/12°, voxel size = 1 mm isotropic; (2) T2-FLAIR: TR/TE/IR = 5000 ms/393 ms/1800 m, voxel size = 1 mm^3^ isotropic; (3) TOF-MRA: TR/TE/FA = 23 ms/3.45 ms/18°, voxel size = 0.2 × 0.2 × 1.5 mm^3^, slice number = 50, saturation band = 60 mm positioned above the imaging slab, scan time = 1 min 26 s. TOF-MRA was performed for vessel segmentation and to determine the appropriate positioning of the PC-MRI scan plane. The imaging coverage extended from the bottom of the pons to the inferior margin of cervical spine C4, ensuring inclusion of the bifurcation while avoiding extra branches; (4) Non-gated PC-MRI was conducted to measure blood flow in the bilateral ICAs and VAs with the following parameters: TR/TE/FA = 20 ms/7 ms/15°, voxel size = 0.45 × 0.45 × 1.5 mm^3^, velocity encoding (VENC) = 60 cm/s. Each artery was scanned for 10 s, totaling 40 s for all measurements. The scan plane was meticulously positioned perpendicular to each artery [i.e., left ICA (LICA), right ICA (RICA), left VA (LVA), and right VA (RVA)], based on TOF-MRA image, simultaneously ensuring it at the center of the field of view (FOV) (see Figure 2A); (5) Pseudo-continuous arterial spin labeling (pcASL) was acquired from 60 subjects to measure cerebral blood perfusion for comparison with total blood flow obtained from four neck arteries measured via PC-MRI. The parameters were as follows: TR/TE/FA = 4120 ms/12.56 ms/120°, voxel size = 3.5 × 3.5 × 4 mm^3^, labeling duration = 1800 ms, single post-labeling delay (PLD) = 2000 ms, with background suppression.

### 2.3 Imaging postprocessing

#### 2.3.1 Vascular segmentation

Vascular segmentation, centerline tracking, and tortuosity metric calculations were performed using custom in-house code executed on MATLAB 2020a (MATLAB and Statistics Toolbox Release 2020a, MathWorks, Inc. Natick, MA). The postprocessing workflow began with defining the region of Interest (ROI) to encompass the arteries. A threshold-based segmentation algorithm was applied to identify arterial pixels based on the signal intensities (Otsu, 1979). Identified pixels from each slice were connected if their faces or edges were adjacent, enabling the reconstruction of 3D rendering of the arteries (see Figure 2B). Following vascular segmentation, arterial centerlines were tracked using the multi-stencils fast marching algorithm (Figure 2C). This method solves the Eikonal equation along predefined stencils to compute the shortest distance from a source point to all other pixels within the image volume (Hassouna and Farag, 2007). The coordinates of the arterial skeleton were extracted and used to calculate geometric variables including inflection count metric (ICM) and ICA angle. The ICM quantifies vessel tortuosity by multiplying the number of turns along the vessel’s course by the ratio of its actual length to the direct length (Klis et al., 2019). The number of turns was determined through visual inspection of the centerline skeleton. For each participant, we first calculated the averaged ICM value for the bilateral ICAs (ICA_ICM) and bilateral VAs (VA_ICM).

To integrate the ICM values from both ICAs and VAs, we proposed an ICM index defined as:


$$I\,C\,M_{i\,n\,d\,e\,x}=0.8\times I\,C\,A\,\_\,I\,C\,M+0.2\times V\,A\,\_\,I\,C\,M,$$

where the weighting factors (0.8 and 0.2) correspond to the intracranial blood supply percentage from the ICAs (80%) and VAs (20%), respectively (Gofur and Bordoni, 2025). The ICA angle is calculated as the angle between the common carotid artery (CCA) and ICA.

#### 2.3.2 PC-MRI postprocessing

PC-MRI data processing was performed following a previously established method (Xu et al., 2009). Initially, preliminary ROIs were manually delineated on the magnitude images by encircling the targeted arteries, specifically the bilateral ICAs and VAs. A signal intensity threshold set at five times the background noise level was applied to the magnitude images, yielding the final vessel mask. These masks were then overlaid on the phase images, allowing for the summation of phase signals (i.e. velocity values) within the mask to determine the blood flow of each artery (blood flow_*ICAs*_ and blood flow_*VAs*_). While the analysis may involve subjective ROI delineation, previous methodological studies have shown that the inter-rater reliability of flow results was relatively high, with an *R*^2^ of 0.994, which is largely attributed to the high flow velocities in these major arteries (Liu et al., 2013). Given that the brain is predominantly supplied by the ICAs and the VAs, the normalized global cerebral blood flow (CBF), expressed in ml/100 g/min, was calculated by summing the blood flow from the four primary feeding arteries and normalizing it to the brain parenchymal mass, which was approximated as 1.06 × parenchymal volume (including both gray and white matter volumes):


$$N\,o\,r\,m\,a\,l\,i\,z\,e\,d\,C\,B\,F=\frac{b\,l\,o\,o\,d\,f\,l\,o\,w_{I\,C\,A\,s}+b\,l\,o\,o\,d\,f\,l\,o\,w_{V\,A\,s}}{1.06\times \left(V\,o\,l_{G\,M}+V\,o\,l_{W\,M}\right)}\,\left(m\,l/100\,g/m\,i\,n\right)$$

The parenchymal volume was derived from T1-weighted structural MRI using FSL (FMRIB Software Library, Oxford University). Further details of the postprocessing steps are described in our previous study (Sun et al., 2022).

#### 2.3.3 PCASL postprocessing

The single post-labeling delay ASL data was processed using a cloud-based tool termed ASL-MRICloud^1^ (Johns Hopkins University, Baltimore, MD). The processing pipeline includes motion correction, calculation of difference image (label – control), determination of equilibrium magnetization (M0), partial volume correction, and CBF quantification (Li et al., 2019). The CBF quantification of CBF follows the consensus guidelines (Alsop et al., 2015):


$$C\,B\,F=\frac{6000\,\lambda \,\Delta \,M\,e^{\frac{P\,L\,D}{T\,1_{b\,l\,o\,o\,d}}}}{2\,\alpha \,T\,1_{b\,l\,o\,o\,d}\,M_{0}\,\left(1-e^{\frac{-\tau }{T\,1_{b\,l\,o\,o\,d}}}\right)},$$

where λ is the brain/blood partition coefficient (0.9 mL/g), Δ*M* is the difference in signal intensity between control and label image, M0 is the signal intensity of the proton density-weighted image, τ is the label duration and α is labeling efficiency (0.85). T1_*blood*_ at 3T is 1650 ms. For each subject, the mean CBF was determined by calculating the average parenchymal CBF across the entire brain, including both gray matter (GM) and white matter (WM).

#### 2.3.4 WMH lesion segmentation

The deep learning-based lesion segmentation method utilized for the automated segmentation of WMH lesions is LST-AI^2^, which integrates 3D T1w and 3D FLAIR images. This method employs an ensemble of three 3D U-Net models that process the skull-stripped, cropped, and intensity-normalized T1w and FLAIR images as input separately (Wiltgen et al., 2024). The final binary lesion map was generated by averaging the three lesion probability maps and subsequent thresholding (with a default threshold of 0.5). Lesions are labeled according to their locations, including periventricular WM (PVWM) and deep subcortical WM (DWM), with WM lesions (WMLs) classified as DWM when they are 13 mm or further from the ventricular surface (Kim et al., 2008). The volume of the lesions was calculated by multiplying the number of segmented voxels by the voxel size of the T_2_-FLAIR imaging.

### 2.4 Evaluation of accuracy and reproducibility of tortuosity metrics

Maximum intensity projection (MIP) images from TOF MRA, obtained in both coronal and sagittal planes, served as a reference to evaluate the accuracy of segmentation. The accuracy of centerline tracking, and the identification of false branches were assessed by overlaying the centerline onto the 3D arterial segmentation. All the segmented arteries and centerline images were visually inspected by 3 raters (Z.S., C.L., and M.M.) with more than 2-year experience. Arteries with incomplete segmentation or cases where the algorithm failed were excluded from further analysis. To further assess reproducibility, a scan-rescan study was performed to account for potential discrepancies related to repositioning errors and TOF-MRA image noise, as described in our previous work (Sun et al., 2022).

### 2.5 Statistical analysis

Data normality of variables, including age, BMI, systolic and diastolic BP, tortuosity and flow measurements were assessed using the Kolmogorov-Smirnov test. For variables that followed a normal distribution, group comparisons between participants with mild and severe lesions were performed using two-tailed independent *t*-tests. For non-normally distributed variables, the Mann-Whitney U test was utilized as an alternative. The chi-square test was conducted to determine if there is a significant association between sex and severity of WMLs. Additionally, independent *t*-tests were also employed to compare the differences in the above-mentioned variables between males and females, allowing for the identification of any sex-related differences. To control for false positives due to multiple comparisons, *p*-values were adjusted using the false discovery rate (FDR) correction.

Partial correlation analyses, controlling for age and sex as covariates, were performed to examine the relationships between blood flow and tortuosity metrics. Specifically, the following associations were examined: (1) normalized CBF versus ICM_*index*_; (2) blood flow in ICAs and ICA_ICM; (3) blood flow in ICAs and ICA angle; and (4) blood flow in VAs and VA_ICM. *P* values were adjusted for multiple comparisons using the FDR correction.

Multiple linear regression analyses were conducted to assess the contribution of various predictors on the WML volumes. Predictors included age, sex, systolic BP, diastolic BP, BMI, and vascular tortuosity metrics (ICM_*index*_ and ICA angle). The regression models were executed separately for different types of WMHs lesion [total WMLs, PVWM lesions (PVWMLs), and DWM lesions (DWMLs)]. The regression model was formulated as follows: WMH lesion ∼ Age + Sex + Systolic BP + Diastolic BP + BMI + ICM_*index*_ + ICA angle. To further explore the relationship between WMLs and global blood supply, partial correlation coefficients were calculated between normalized CBF and total volume of WMLs, as well as between pcASL-CBF and total volume of WMLs, while adjusting for age and sex as covariates.

To determine whether normalized CBF derived from PC-MRI can substitute for CBF measured using pcASL MRI, the association between these two CBF measurements was evaluated using Pearson’s correlation coefficient. Subsequently, relationships between tortuosity metrics and each of the two CBF measurements were assessed using partial correlations controlling for age and sex. All statistical analyses were performed using SPSS version 29 and Prism 10. A significance threshold of *p* < 0.05 was applied unless otherwise specified.

## 3 Results

### 3.1 Tortuosity and flow comparisons in mild and severe lesion groups

The final analysis included 75 subjects (mean age: 73.1 ± 5.5 years; 42 females/33 males). Among them, 39 had mild WMH lesions (mean age: 71.1 ± 4.7 years, 17 females/22 males) and 36 had severe lesions (mean age: 75.1 ± 5.6 years, 25 females/11 males). A Chi-square analysis revealed a significant sex difference in WMH severity, with females exhibiting higher WMH burden (χ^2^ = 5.09, *p* = 0.024). After FDR correction for multiple comparisons, no significant group differences were observed in vascular risk factors, including BMI, systolic BP, and diastolic BP.

Independent *t*-tests showed that subjects with severe WMHs had a significantly higher ICM_*index*_ (*p* < 0.001) and larger ICA angle (*p* = 0.003) compared with those in the mild group subjects. Additionally, normalized CBF measured using PC-MRI was significantly lower in the severe WMH group (*p* = 0.03), whereas pcASL-derived CBF did not differ between groups (*p* = 0.30). Detailed descriptive statistics and group comparison results are summarized in Table 1, with significant findings marked by asterisks. Tortuosity and flow measurements of bilateral ICAs and VAs were also analyzed separately and compared between groups (Supplementary Table 1).

**TABLE 1:** Data summary of two different lesion groups (mean ± standard deviation).

Variables	All participants (*n* = 75)	Mild group (*n* = 39)	Severe group (*n* = 36)	*p*-value
Age	73.1 ± 5.5	71.1 ± 4.7	75.1 ± 5.5	0.02*
Sex (F/M)	42/33	17/22	25/11	0.02*
Systolic BP (mmHg)	131.1 ± 16.4	128.3 ± 17.7	134.2 ± 14.6	0.18
Diastolic BP (mmHg)	70.4 ± 11.8	68.1 ± 12.0	73.0 ± 11.4	0.19
BMI	25.6 ± 4.5	25.3 ± 4.0	25.98 ± 5.0	0.62
**Number of subjects for:**				
BP measurements	65	34	31	
BMI measurements	75	39	36	
PC-MRI	75	39	36	
pcASL	60	29	31	
Lesion volume (cm^3^)	5.37 ± 7.52	0.90 ± 1.15	10.21 ± 8.47	<0.001*
ICM_index_	6.16 ± 2.06	5.08 ± 1.72	7.33 ± 1.74	<0.001*
ICA angle (degree)	56.35 ± 14.96	50.41 ± 13.67	62.96 ± 13.64	0.003*
Normalized CBF (ml/100 g/min)	39.53 ± 7.52	42.14 ± 8.07	36.71 ± 5.77	0.03*
pcASL CBF (ml/100 g/min)	38.52 ± 10.22	39.21 ± 10.26	36.88 ± 9.17	0.30

The FDR adjusted *p* < 0.05 was set as threshold of statistical significance.

*Indicates statistically significant.

### 3.2 Tortuosity and flow comparisons in male and female groups

Males and females had similar ICM_*index*_ (*p* = 0.99). However, females showed significantly greater ICA angle and VA_ICM values compared with males (both *p* = 0.03). Although ICA_ICM values were also higher in females than in males, the difference did not reach statistical significance (*p* = 0.25). Females exhibited significantly larger total WMH volumes than males (*p* = 0.002). Regarding CBF, females demonstrated slightly higher pcASL-derived CBF values than males (*p* = 0.046), whereas normalized CBF did not differ significantly between groups (*p* = 0.48). Detailed results of these comparisons are provided in Table 2.

**TABLE 2:** Data summary of male and female individuals (mean ± standard deviation).

Variables	Male (*n* = 33)	Female (*n* = 42)	*p*-value
Lesion volume (cm^3^)	3.81 ± 7.96	6.59 ± 7.00	0.002*
ICM_index_	6.16 ± 2.14	6.16 ± 2.01	0.99
ICA_ICM	6.23 ± 2.68	6.47 ± 2.34	0.25
VA_ICM	4.32 ± 2.44	7.20 ± 3.63	0.03*
ICA angle (degree)	51.13 ± 14.38	59.38 ± 15.91	0.03*
Normalized CBF (ml/100 g/min)	39.20 ± 8.03	40.55 ± 7.95	0.48
pcASL CBF (ml/100 g/min)	36.67 ± 11.04	41.08 ± 10.26	0.046*

The FDR adjusted *p* < 0.05 was set as threshold of statistical significance.

*Indicates statistically significant.

### 3.3 Tortuosity in relation to flow measurements

**FIGURE 3:**
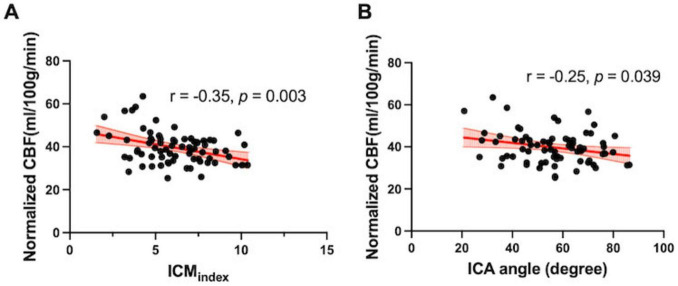
Association between tortuosity and flow measurements. **(A)** ICM_index_ and **(B)** ICA angle was negatively correlated with normalized global CBF quantified from the PC-MRI (*r* = –0.35, *p* = 0.003; *r* = –0.25, *p* = 0.04, respectively).

After adjusting for age and sex, partial correlation analyses revealed significant negative associations between ICM_*index*_ and normalized CBF (*r* = −0.35, *p* = 0.003); as well as between ICA angle and normalized CBF (*r* = −0.24, *p* = 0.04) (Figures 3A, B).

Similar trends were observed when ICAs and VAs were analyzed separately. ICA blood flow showed an inverse correlation with ICA_ICM (*r* = −0.40, *p* < 0.001) and a weaker negative correlation with ICA angle (*r* = −0.27, *p* = 0.02) (Supplementary Figures 1A, B). However, no correlation was observed between VA_ICM and VA blood flow (*r* = −0.007, *p* = 0.95) (see Supplementary Figure 1C).

### 3.4 Tortuosity and Flow Measurements in Relation to the WMH Lesions

**FIGURE 4:**
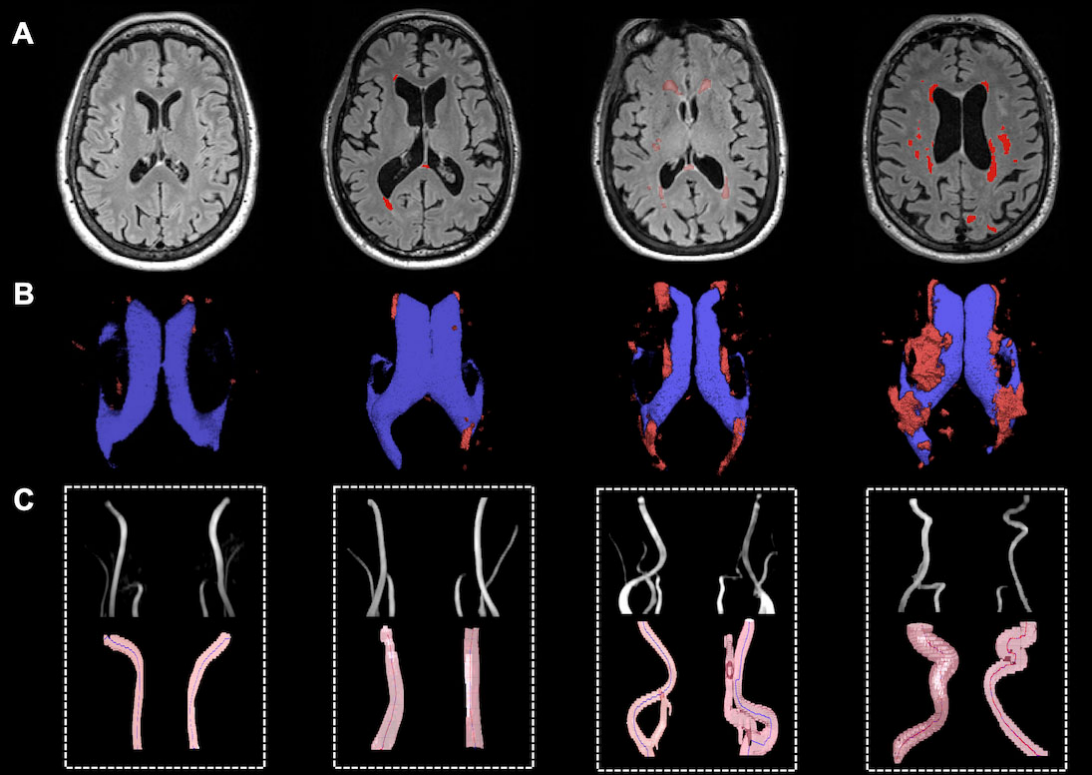
Representative illustration of WMH lesions and tortuous ICAs and VAs. **(A)** WMH lesion on FLAIR MRI; **(B)** Lateral ventricle (blue) and WM lesion segmentations (red); **(C)** Vascular segmentation and centerline tracking showed subjects with higher lesion load had higher tortuosity level.

Qualitative analysis indicated that subjects with more tortuous neck brain-feeding arteries tended to exhibit a higher WML load (Figure 4). Multiple linear regression was further conducted to examine the relationship between tortuosity metrics and lesion volumes across different WML categories. The regression model for total WML volume explained 30.6% of the variance (*r*^2^ = 0.31, adjusted *r*^2^ = 0.22; F(7, 56) = 3.53, *p* = 0.003). For PVWMLs, the regression model explained 32.7% of the variance (*r*^2^ = 0.33, adjusted *r*^2^ = 0.24; F(7, 56) = 3.88, *p* = 0.002). In contrast, the model for DWMLs did not reach statistical significance (*r*^2^ = 0.20, adjusted *r*^2^ = 0.10, F(7, 56) = 2.03, *p* = 0.07). Table 3 summarizes the regression coefficients (β) and their significance levels for each lesion category.

**TABLE 3:** Effects of age, sex, vascular risk factors, and vascular tortuosity on WMHs.

Variables	Coefficient (β) (95%CI)	Standardized coefficient	*p*-value
**Total WM lesions**			
Age	0.399 (0.062, 0.737)	0.290	0.021*
Sex	−1.77 (−5.338, 1.798)	−0.118	0.325
Systolic BP (mmHg)	−0.028 (−0.152, 0.095)	−0.062	0.648
Diastolic BP (mmHg)	0.052 (−0.138, 0.242)	0.075	0.583
BMI	0.206 (−0.193, 0.605)	0.121	0.305
ICM_index_	1.204 (0.319, 2.090)	0.329	0.009*
ICA angle	0.051 (−0.075, 0.177)	0.102	0.418
**PVWM lesions**			
Age	0.331 (0.058, 0.605)	0.293	0.018*
Sex	−1.660 (−4.547, 1.226)	−0.134	0.254
Systolic BP (mmHg)	−0.008 (−0.109, 0.092)	−0.023	0.866
Diastolic BP (mmHg)	−0.008 (−0.161, 0.146)	−0.013	0.922
BMI	0.086 (−0.237, 0.409)	0.061	0.594
ICM_index_	1.054 (0.337, 1.770)	0.351	0.005*
ICA angle	0.039 (−0.063, 0.141)	0.094	0.448
**DWM lesions**			
Age	0.070 (−0.038, 0.179)	0.054	0.200
Sex	−0.062 (−1.208, 1.084)	0.572	0.914
Systolic BP (mmHg)	−0.024 (−0.064, 0.016)	0.020	0.236
Diastolic BP (mmHg)	0.077 (0.016, 0.137)	0.030	0.015*
BMI	0.118 (−0.010, 0.246)	0.064	0.071
ICM_index_	0.138 (−0.147, 0.422)	0.142	0.337
ICA angle	0.014 (−0.026, 0.055)	0.020	0.489

*Indicates statistically significant.

**FIGURE 5:**
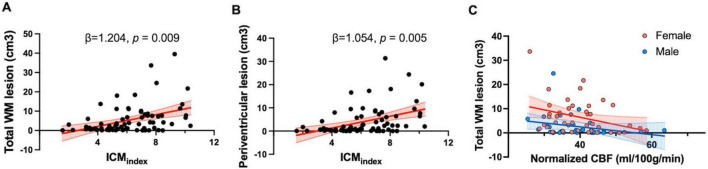
Tortuosity and flow measurements in relation to the WMH lesion. **(A)** Scatter plot showing a positive correlation between total WM lesion volume and ICM_index_ (β = 1.204, *p* = 0.009). **(B)** Scatter plot showing a positive association between ICM_index_ and periventricular WM lesion volume (β = 1.054, *p* = 0.005); **(C)** Partial correlation analyses showed a negative correlation between normalized CBF and total WM lesion volume (*r* = –0.27, *p* = 0.018). Different regression slopes were found for males (*Y* = –0.16X + 8.91) and females (*Y* = –0.31X + 18.67), indicating sex-specific differences in the relationship.

Specifically, the ICM_*index*_ showed a significant positive association with total WML volume (β = 1.204; 95% CI: 0.319–2.090; *p* = 0.009) and PVWMLs (β = 1.504; 95% CI: 0.337–1.770, *p* = 0.005) (Figures 5A, B). However, ICM_index_ was not significantly associated with DWMLs (*p* = 0.34). Additionally, ICA angle was not significantly related to lesion volumes in any category (total WMLs: *p* = 0.42; PVWMLs: *p* = 0.45; DWMLs: *p* = 0.49).

Partial correlation analyses were conducted to examine the relationship between normalized CBF measured by PC-MRI and total WML volume, controlling for age and sex. A significant negative partial correlation was observed (*r* = −0.27, *p* = 0.018), indicating the lower normalized CBF was associated with a higher lesion burden. Furthermore, sex-specific regression analyses demonstrated different slopes for males (*Y* = −0.16X + 8.91) and females (*Y* = −0.31X + 18.67), suggesting greater sensitivity of females to reductions in blood supply (Figure 5C). However, there was no significant association between pcASL-derived CBF and total WML volumes (*p* = 0.27).

### 3.5 Relationship between normalized CBF derived from PC-MRI and pcASL-MRI

Normalized global CBF measured using PC-MRI showed a strong correlation with global CBF obtained via pcASL (*r* = 0.63, *p* < 0.001), suggesting that cerebral perfusion measured by pcASL is substantially influenced by bulk flow from large brain-feeding arteries, despite the two techniques reflecting different hemodynamic parameters. In contrast to the negative correlations observed between PC-MRI-derived normalized CBF and tortuosity metrics (ICM_index_ and ICA angle, as mentioned in section “3.3 Tortuosity in relation to flow measurements”), pcASL-derived CBF did not show significant associations with tortuosity metrics (ICM_index_: *p* = 0.49; ICA angle: *p* = 0.09). This differential pattern implies that PC-MRI and pcASL may differ in their sensitivity to extracranial vascular geometry, with PC-MRI-derived CBF being more reflective of upstream arterial morphology.

### 3.6 Accuracy and reproducibility evaluation

Regarding the accuracy evaluation of quantitative analysis of large artery tortuosity, the algorithm successfully extracted vascular skeleton for 70 subjects’ ICAs, yielding a subject-based successful rate of 93.3%. Further investigation revealed that satisfactory arterial segmentation could be obtained by selecting an appropriate intensity threshold using multi-level algorithms. Failures in the centerline tracking algorithm were likely due to the following reasons: (1) complex arterial geometry, such as coiling, where overlapping segments interfered with tracking; (2) proximity of the ICA to the external carotid artery (ECA), leading to misidentification of the ECA as part of ICA. The tracking failures could potentially be mitigated by increasing the signal threshold, which would help exclude unwanted vessels and reduce segment overlap.

Among the test–retest reproducibility scans (4 males, 3 females; mean age 26.4 ± 3.7 years), the algorithm successfully extracted the vascular skeleton for all arteries with no failures. The mean and standard error of inter-session coefficient of variation (CoV) for ICM measurements were 3.0% ± 0.76% across all 28 arteries (Sun et al., 2022). The reproducibility of PC–MRI blood flow quantification have been previously reported by Peng et al. (2015) and Liu et al. (2013, 2014), with inter-session CoV of 5.25 ± 2.93% for blood flow and 7.41 ± 2.99% for normalized global CBF.

## 4 Discussion

In this study, we employed a semi-automatic pipeline for vascular reconstruction and quantitative tortuosity analysis to examine the relationship between extracranial arterial tortuosity, cerebral blood flow, and WMH burden. We found that ICAs and VAs exhibited greater tortuosity in the severe WMH group compared to the mild lesion group, consistent with previous studies (Chen et al., 2020; Zhang et al., 2019; Koge et al., 2022). Tortuosity metrics, such as ICM_index_ and ICA angle, were negatively associated with PC-MRI-derived normalized CBF from major extracranial brain-feeding arteries. To account for the integrative role of the circle of Willis, which facilitates communication between anterior and posterior circulations, we used a weighted index combining ICAs and VAs according to their supply contribution. Unlike earlier studies that relied on categorical classifications, we applied quantitative metrics for both WMHs and tortuosity, enabling more precise correlation analysis. These vascular geometric changes may serve as early markers of vascular degeneration and predictors of progression to advanced pathology, such as atherosclerotic plaque formation and stenosis (Bir and Kelley, 2022), offering potential for earlier diagnosis and intervention.

Vascular tortuosity in older adults likely results from vessel wall remodeling due to chronic and repetitive pulsatile blood flow (Frosen et al., 2019; Chien, 2008), leading to sluggish blood flow and abnormal wall shear stress. We observed an inverse correlation between tortuosity and blood flow, likely reflecting increased vascular resistance (Li et al., 2012; Xie et al., 2013). Twisted arterial segments and large ICA angle were associated with disturbed or low wall shear stress (Markl et al., 2010), which can impair endothelial function and promote atherosclerosis (Hakim et al., 2023). However, this association was evident in ICAs but not in VAs (see Supplementary Figure 1). While prior studies link basilar artery tortuosity to posterior circulation deficits (Szalontai et al., 2021; Zhang et al., 2019), the relationship between VA geometry and blood flow remains unclear. Unlike ICA, VA course is structurally constrained by the cervical spine foramens, which may limit the impact of vessel wall remodeling and large-scale geometric alterations (Burle et al., 2022). This constraint may explain the lack of association between VA geometry and VA blood flow. Further studies are warranted to clarify the contribution of vertebrobasilar geometry to circulation and WMH development.

The link between age-related extracranial brain-feeding artery changes and WMH severity remains debated. While some studies implicate that ICA stenosis impairs cerebral perfusion and contributes to WMH progression (Chutinet et al., 2012), others emphasize the role of vascular risk factors such as age, hypertension, and glucose levels (Potter et al., 2012). In our regression analyses, age and ICM_index_ have demonstrated and emerged as predictors of total WMH volume. When WMHs were subdivided, this association was observed only for PVWMLs, while DWMLs were instead associated with diastolic blood pressure. The distinct vascular anatomy and pathogenic mechanisms of the PVWM and DWM regions likely underlie these differences (Wu et al., 2021).

PVWMLs occur in regions mainly supplied by non-anastomosing branches of the middle cerebral artery. By contrast, DWMLs are supplied by medullary penetrating branches from the main pial arteries, making it less sensitive to hypoperfusion but more prone to small vessel disease and fibrohyalinosis, particularly in hypertensive individuals (Brown et al., 2009). Hypertension may impair autoregulation, increasing tortuosity and reducing the adaptability of medullary arteries (Moody et al., 1991; Akashi et al., 2017). The specific role diastolic BP in DWML development remains unclear and warrants further study in larger cohorts with detailed medication data. Overall, the distinct vascular supply and vulnerability of PVWM and DWM regions help explain their differing associations with arterial tortuosity and blood pressure. While ICA angle was not strongly associated with WMHs, a weak positive trend suggests that larger angles may contribute to greater lesion burden. This aligns with prior studies linking disturbed wall shear stress at bifurcations to endothelial dysfunction and stenosis (Flaherty et al., 2013).

We quantified global CBF using PC-MRI by summing flow from the bilateral ICAs and VAs, normalized to brain parenchyma mass. PC-MRI–derived global CBF correlated well with pcASL-derived CBF, although values were consistently higher, likely reflecting PC-MRI overestimation from partial volume effects and pcASL underestimation from imperfect labeling efficiency (Dolui et al., 2016). Variations in brain tissue density, blood T1, and the brain partition coefficient may also contribute to this discrepancy. Notably, PC-MRI–derived CBF was significantly associated with tortuosity metrics and WMH volume, whereas pcASL-derived CBF showed no such associations. This divergence underscores the complementary nature of the two approaches: PC-MRI directly measures large-vessel inflow to the brain, providing a robust index of global bulk flow, while pcASL estimates regional tissue perfusion, capturing microvascular function but with lower SNR and greater sensitivity to post-labeling delays, particularly in older adults (Dai et al., 2017). Moreover, pcASL-estimates may be influenced by intracranial arterial branching patterns and collateral flow.

Despite these methodological differences, prior work has shown that intracranial ICA and MCA flow measured with PC-MRI positively correlates with pcASL-derived perfusion in cortical regions from the postcentral gyrus, superior temporal gyrus, and frontal lobes (Clark et al., 2017). These findings suggest that extracranial tortuosity, by altering distal flow and promoting age-related vascular remodeling (Han, 2012), may exert downstream effects on both global inflow and regional perfusion.

We also found sex-based differences in vascular tortuosity, lesion volume, and cerebral perfusion. Women, particularly post-menopausal, showed a higher WMH burden (Lohner et al., 2022) despite similar CBF levels compared with men. Given that WMHs are more strongly linked to cognitive decline, executive dysfunction, memory loss, and impaired daily activities in women than in men, these findings align with prior studies suggesting that females may be more vulnerable to hypoperfusion-related brain changes (Arenaza-Urquijo et al., 2024), possibly due to hormonal shifts post-menopause. These sex-specific vulnerabilities warrant further investigation.

This study has several limitations. First, our analysis focused on extracranial ICAs and VAs and did not include intracranial arteries such as the MCAs or collateral flow within the circle of Willis. These intracranial vessels are more challenging to evaluate without high-resolution, contrast-enhanced imaging. Since the purpose of this study was to establish a fast, clinically feasible protocol, we prioritized extracranial arteries, which can be reliably captured using non-contrast MRA. Second, we lacked complete data on vascular risk factors (e.g., smoking, glucose, lipids) and cognitive measurements such as Mini-Mental State Examination (MMSE). In this study, we adjusted for BP and used BMI as a proxy for hyperlipidemia. Future studies should incorporate standardized cognitive assessments to clarify their relationship with vascular parameters and WMH burden. Third, PC-MRI provides only global CBF estimates and does not offer territory-specific perfusion information, which may limit its ability to establish spatial relationships with intracranial lobar regions. Finally, while our rapid MRA protocol was efficient, it may miss subtle vessel wall details or diameter changes; thus, we interpreted flow variations primarily in relation to geometry rather than wall pathology.

## 5 Conclusion

Our study highlights the impact of geometric abnormalities in large neck arteries on WMH development, linking vascular tortuosity to compromised cerebral blood supply. Using a clinical available TOF- and PC-MRA protocol combined with a robust post-processing pipeline, we demonstrate the potential of quantitative vascular tortuosity metrics as extracranial markers of WMH severity and neuronal function. These findings emphasize the critical role of age-related vascular geometry changes in assessing cerebrovascular health and its broader implications for cognitive function.

## Data Availability

The original contributions presented in this study are included in this article/Supplementary material, further inquiries can be directed to the corresponding author.

## Footnotes

1
www.MRICloud.org

2
https://github.com/CompImg/LST-AI
