# Supplementary material for: Tortuous extracranial arteries contribute to white Matter hyperintensities in aging brains
Source: Front Aging Neurosci. 2025 Oct 9;17:1641214. doi: 10.3389/fnagi.2025.1641214 (PMC12546081; doi:10.3389/fnagi.2025.1641214)
Supplement: Supplementary file 1 [file Table_1.docx]

Supplementary Material

# Supplementary Tables and Figures

## Supplementary Table

**Supplementary Table 1**. Data summary of tortuosity metrics and flow measurements in ICAs and VAs of different lesion groups (mean± standard deviation).

|  | **All participants**  **(n = 75)** | **Mild group**  **(n = 39)** | **Severe group**  **(n = 36)** | ***P*-value** |
| --- | --- | --- | --- | --- |
| **ICA measurements** |  |  |  |  |
| ICA-ICM | 6.28±2.40 | 5.33±2.12 | 7.30±2.29 | 0.003* |
| ICA angle (degree) | 56.35±14.96 | 50.41±13.67 | 62.96±13.64 | 0.003* |
| ICA area (cm^2^) | 0.155±0.03 | 0.16±0.03 | 0.15±0.03 | 0.25 |
| ICA blood flow (ml/min) | 358.2±66.12 | 388.8±67.40 | 325.1±46.23 | <0.001* |
| **VA measurements** |  |  |  |  |
| VA-ICM | 5.68±3.65 | 4.05±2.70 | 7.45±3.76 | <0.001* |
| VA area (cm^2^) | 0.068±0.02 | 0.069±0.02 | 0.068±0.02 | 0.98 |
| VA blood flow (ml/min) | 107.2±32.96 | 108.8±35.1 | 105.3±30.93 | 0.61 |

## Supplementary Figure


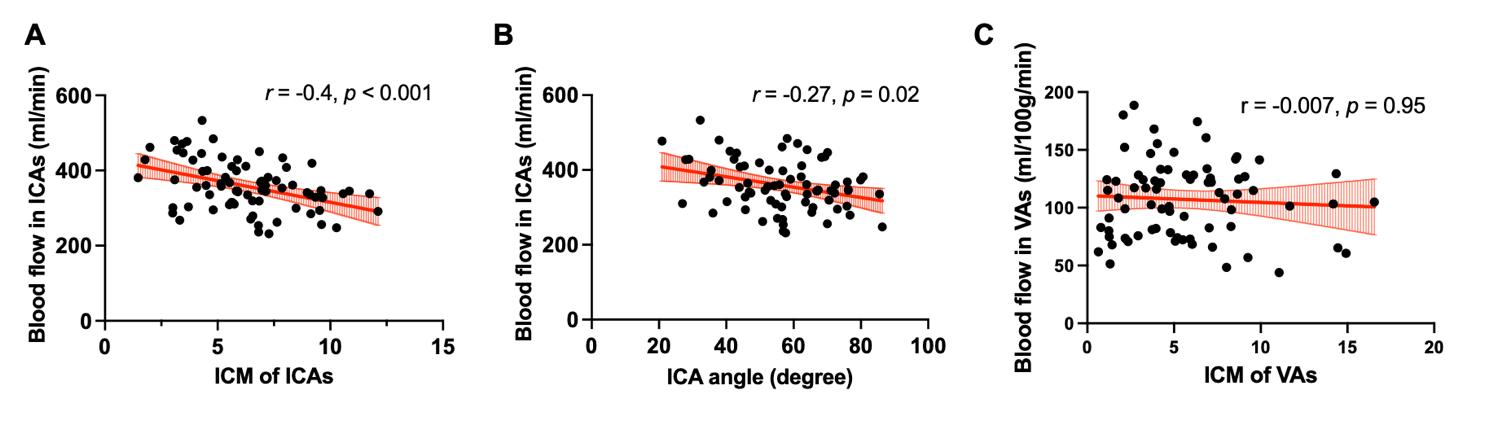


**Supplementary Figure 1.** **Association between vascular tortuosity and flow measurements in ICAs and VAs. (A)** ICA-ICM and **(B)** ICA angle was negatively correlated with the blood flow within the ICAs (*r* = -0.4, *p* < 0.001; *r* = -0.27, *p* = 0.02, respectively). (C) There was no significant correlation between ICM of VAs and blood flow in VAs (*p =* 0.95).
